# Axonal Dysfunction Precedes Motor Neuronal Death in Amyotrophic Lateral Sclerosis

**DOI:** 10.1371/journal.pone.0158596

**Published:** 2016-07-06

**Authors:** Yuta Iwai, Kazumoto Shibuya, Sonoko Misawa, Yukari Sekiguchi, Keisuke Watanabe, Hiroshi Amino, Satoshi Kuwabara

**Affiliations:** Department of Neurology, Graduate School of Medicine, Chiba University, Chiba, Japan; University of Sydney, AUSTRALIA

## Abstract

Wide-spread fasciculations are a characteristic feature in amyotrophic lateral sclerosis (ALS), suggesting motor axonal hyperexcitability. Previous excitability studies have shown increased nodal persistent sodium conductances and decreased potassium currents in motor axons of ALS patients, both of the changes inducing hyperexcitability. Altered axonal excitability potentially contributes to motor neuron death in ALS, but the relationship of the extent of motor neuronal death and abnormal excitability has not been fully elucidated. We performed multiple nerve excitability measurements in the median nerve at the wrist of 140 ALS patients and analyzed the relationship of compound muscle action potential (CMAP) amplitude (index of motor neuronal loss) and excitability indices, such as strength-duration time constant, threshold electrotonus, recovery cycle and current-threshold relationships. Compared to age-matched normal controls (n = 44), ALS patients (n = 140) had longer strength-duration time constant (SDTC: a measure of nodal persistent sodium current; p < 0.05), greater threshold changes in depolarizing threshold electrotonus (p < 0.05) and depolarizing current threshold relationship (i.e. less accommodation; (p < 0.05), greater superexcitability (a measure of fast potassium current; p < 0.05) and reduced late subexcitability (a measure of slow potassium current; p < 0.05), suggesting increased persistent sodium currents and decreased potassium currents. The reduced potassium currents were found even in the patient subgroups with normal CMAP (> 5mV). Regression analyses showed that SDTC (R = -0.22) and depolarizing threshold electrotonus (R = -0.22) increased with CMAP decline. These findings suggest that motor nerve hyperexcitability occurs in the early stage of the disease, and precedes motor neuronal loss in ALS. Modulation of altered ion channel function could be a treatment option for ALS.

## Introduction

From the date of Charcot, pathomechanisms of upper and lower motor neuron degeneration in amyotrophic lateral sclerosis (ALS) have not been fully elucidated [1]. Probably, multiple pathomechanisms underlie the development of motor neuron death, with motor neuronal hyperexcitability potentially contributing to it [2]. Wide-spread fasciculations are a specific feature of ALS and suggest motor nerve terminal hyperexcitability [3,4]. Actually, in ALS motor axons, increased sodium and decreased potassium currents, both of these inducing hyperexcitability, have been reported [5–8]. Glutamate is the main excitatory neurotransmitter in the central nervous system, and excessive glutamate induces neurodegeneration, known as excitotoxicity [9,10]. Futhermore, ALS motor neuron has decreased capacity for Ca^2+^ influx [11]. As such, motor neuronal hyperexcitability appears to increase exposure to glutamate, Ca^2+^ influx and result in motor neuronal degeneration. Riluzole is known to lower the concentration of glutamate in the synaptic cleft and support this hypothesis [12].

While the association of motor neuronal hyperexcitability with motor neuron death has been gradually revealed, the relationship of the extent of motor axonal loss and axonal hyperexcitability has not been fully elucidated. Prior study followed up nerve excitability findings in 37 ALS patients during 3 months, relatively short period, and concluded that potassium currents decrease along with disease progression [13], although the onset of nerve hyperexcitability and long term alterations remain unknown. Briefly, it is not proven whether excitability alteration precedes onset of axonal loss and how excitability changes during long periods of time. If excitability alteration precedes the onset of axonal loss, it may suggest that hyperexcitability is relatively upstream of motor neuronal degeneration process.

To disclose the association of the extent of motor neuronal death with axonal hyerexcitability, we cross-sectionally performed nerve excitability testing in over 100 ALS patients and analyzed these.

## Materials and Methods

### Subjects

Consecutive patients with ALS were seen at Chiba University Hospital from January 2001 through to September 2014 and were included into this study. The following patients were excluded: patients with a family history of motor neuron disease, genetically proven hereditary ALS, patients with concomitant disease which affects peripheral nerve, such as diabetes mellitus and carpal tunnel syndrome, patients with severe axonal loss in the median nerve, in which we could not perform excitability testing or patients who took riluzole at the time of testing. MRI and conventional nerve conduction study, including sensory nerve conductin study, were performed to exclude these diseases. Patients fulfilled the revised El Escorial criteria for definite or probable ALS [14]. Of these patients, 37 patients were included into the previous study [5]. Control data for nerve excitability measurements were obtained from 44 age- and gender-matched normal controls (22 men; age, 51 to 86 years, mean 64.2 years). None of them had clinical or electrophysiological evidence of a peripheral nerve or lower motor neuron disorder. All subjects gave verbal informed consent, and the contents of oral informed consent were recorded in medical records. We did not obtain written informed consent from all participants, because this was a retrospective study.This study was approved by the Chiba University institutional review committees. Approval from an ethical standards committee to conduct this study was received.

### Nerve conduction study

Standard nerve conduction study was performed in all patients to exclude differential diagnosis. CMAP amplitude in abductor pollicis brevis (APB) muscle was picked up for analyses.

### Multiple Excitability Measurements

Multiple excitability properties were measured for the median nerve at the wrist and recorded from abductor APB muscle, (QTRAC with multiple protocol TRONDXM2 or NF, Institute of Neurology, London, UK), as reported elsewhere [6,7,15]. Skin temperature near the stimulus site was maintained > 32°C. The following excitability indices were measured and included; strength-duration time constant (SDTC), threshold electrotonus (TE), refractoriness, superexcitability, and late subexcitability of the recovery cycle of axonal excitability with a single supramaximal conditioning stimulus and current-threshold relationship. SDTC was calculated from the relationship between stimulus intensity and duration to evoke a target potential, using the formulation of Weiss [16–18]. In TE, a 100ms sub-threshold polarizing pulse was delivered as a conditioning stimulus, and threshold change to produce a target CMAP response (40% maximus) was measured. The recovery cycle was recorded as the recovery of axonal membrane excitability following a supramaximal conditioning stimulus. A current-threshold relationship was obtained by tracking threshold changes, following a sub-threshold 200ms polarizing current.

Combinations of these parameters reflect channel function and membrane potential in axons [16,17]. SDTC is sensitive to persistent sodium conductances. Fast pottasium currents are estimated mainly by TEd (10-30ms), superexcitability and depolarizing current in current-threshold relationship. TEd (90-100ms), late subexcitability and depolarizing current in current-threshold relationship mainly represent slow potassium currents.

### Statistical Analysis

All statistical tests were two-sided. To compare total ALS cohort and normal controls, Mann Whitney U test or Fisher’s exact test were performed. In subanalysis, ALS patients were divided into two groups according to CMAP amplitudes in APB muscle. CMAP cut-off value was set as 5mV because this is normal cut-off value of our laboratory, measured in 101 normal controls. In this analysis, Dunnett test was executed.

To examine the relationship between CMAP amplitude and nerve excitability indices, Pearson’s correlation coefficient was used.

Data are presented as mean ± S.E. The level of statistical significance was established at *P <* 0.05. Also in regression analyses, *P <* 0.05 was judged as significant relationship [19]. If |R| was over 0.2, those parameters were judged to be correlated to each other. All statistical analyses were performed by SPSS Statistics version 22 software.

## Results

### Clinical profiles

One hundred forty patients who met the inclusion criteria were enrolled. Clinical profiles in ALS patients are shown in Table 1. Their mean age was 66.6 ± 0.8 years old, and 55% were male. These were not significantly different from the control group. Mean disease duration was 16.5 ± 1.2 months, and first symptom affected the limb regions in 69% patients. Mean CMAP was 4.5 ± 0.3 mV.

**Table 1:** Clinical and neurophysiological profiles in 140 ALS patients.

				Amyotrophic lateral sclerosis			Normal (n = 44)
				All (n = 140)	CMAP > 5mV (n = 53)	CMAP < 5mV (n = 87)	
Clinical profiles							
	Age at assessment (year)			66.6 (0.8)	64.6 (1.2)	67.8 (1.1)	64.2 (1.4)
	Gender (male: female)			77: 63	29: 24	48: 39	22: 22
	Disease duration (month)			16.5 (1.2)	13.8 (1.2)	18.1 (1.7)	
	Site of onset (bulbar; arm; leg)			43; 49; 48	21; 9; 23	22; 40; 25	
Nerve conduction study							
	CMAP amplitude (Median nerve)		(mV)	4.5 (0.3)	7.8 (0.3)	2.5 (0.1) *	7.0 (0.4)
Nerve excitability testing							
	Strength-duration time constant		(ms)	0.48 (0.01)*	0.45 (0.01)	0.50 (0.02)*	0.43 (0.01)
	Threshold electrotonus						
		TEd (10–30 ms)	(%)	71.3 (0.9)*	70.0 (0.7)	72.1 (1.4)*	66.1 (0.9)
		TEd (90–100 ms)	(%)	52.2 (0.8)*	49.6 (0.8)*	53.8 (1.2)*	44.5 (0.9)
		TEh (90–100 ms)	(%)	-129.8 (2.3)	-130.9 (3.0)	-129.2 (3.2)	-120.7 (4.0)
	Recovery cycle						
		Refractoriness	(%)	20.4 (2.7)	18.5 (2.8)	21.6 (4.1)	13.3 (3.7)
		Superexcitability	(%)	-28.6 (0.7)*	-28.2 (0.8)*	-28.9 (1.0)*	-21.0 (1.2)
		Late subexcitability	(%)	13.0 (0.5)*	14.0 (0.8)	12.4 (0.7)*	16.3 (0.7)
	Current threshold relationship						
		40% depolarizing current	(%)	51.9 (0.9)*	48.9 (0.7)*	53.6 (1.4)*	43.6 (1.0)
		100% hyperpolarizing current	(%)	-302.0 (6.6)	-312.4 (8.2)	-295.8 (9.3)	-299.6 (8.6)

ALS = amyotrophic lateral sclerosis; CMAP = compound muscle action potential; TE = threshold electrotonus; Data are given as mean (SE).

* P < 0.05, compared with normal values

### Excitability alterations in ALS cohort

**Fig 1:**
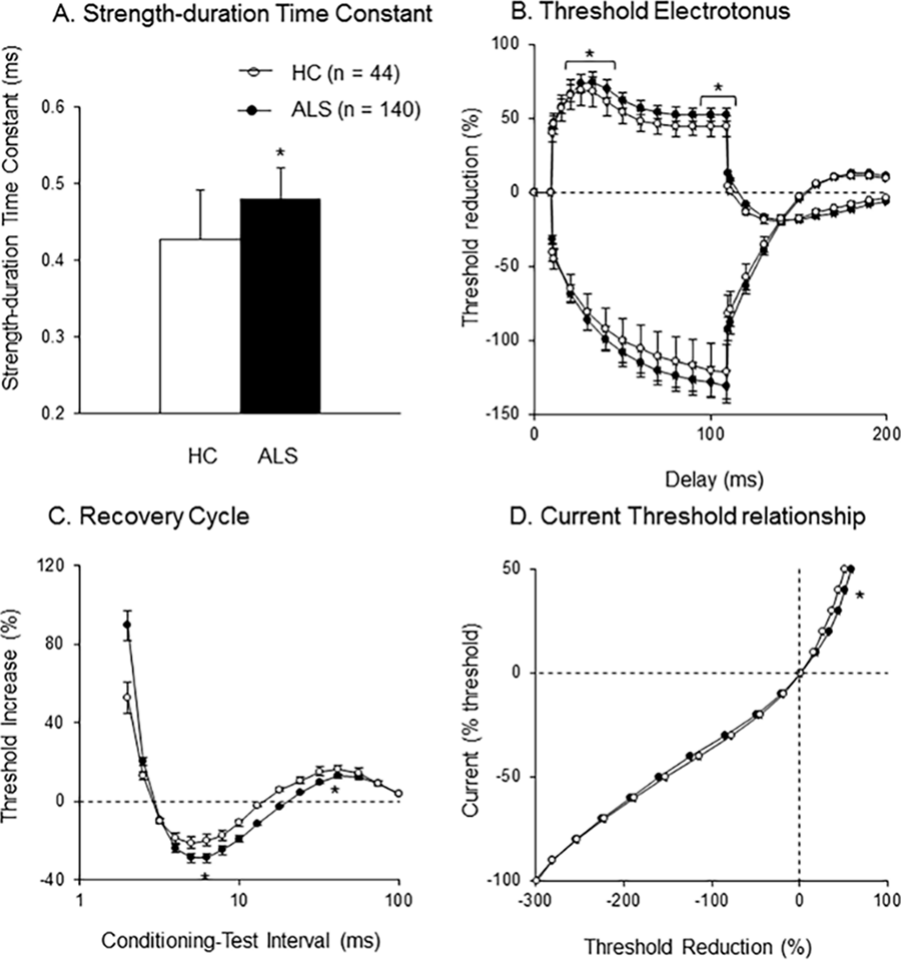
Nerve excitability indices in healthy control and ALS. Averaged excitability indices in normal controls (NC) (n = 44) and total amyotrophic lateral sclerosis (ALS) cohort (n = 142). Compared to NC, significant greater strength-duration time constant (SDTC) (*p < 0.05) (A), greater depolarizing threshold electrotonus (TEd) (TEd 10-30ms and TEd 90-100ms; *p < 0.05) (B), increased superexcitabilty (*p < 0.05) and reduced late subexcitability (*p < 0.05) in recovery cycle (C) and increased 40% depolarizing currents in current threshold relationship (I/V) (*p < 0.05) (D) were found in amyotrophic lateral sclerosis (ALS) patients. Data are given as mean ± SE.

Results of multiple excitability measurements are shown in Table 1, Fig 1 and S1 Table. Compared to normal subjects, significantly longer SDTC, greater depolarizing threshold electrotonus (TEd), greater superexcitability and reduced late subexcitability in recovery cycle and greater reduction of threshold in 40% depolarizing current in current threshold relationship were demonstrated in the total ALS cohort (n = 140). These findings suggest increased persistent sodium conductances and decreased fast and slow potassium currents.

Also in the subgroup analysis, ALS patients with under 5mV CMAP amplitude had similar findings. Significantly longer SDTC, greater TEd, greater superexcitability and reduced late subexcitability in recovery cycle and greater reduction of threshold in 40% depolarizing current in current threshold relationship were found in this ALS cohort (n = 87) (Table 1). ALS patients with over 5mV CMAP amplitude demonstrated similar findings, although extents of these differences were less evident, and SDTC did not reach significant difference. These results suggest that decreased potassium currents are obvious even in patients with preserved CMAP amplitudes, and increased sodium and decreased potassium currents were more prominent in patients with severe axonal loss.

### Association of excitability indices with CMAP amplitude

**Fig 2:**
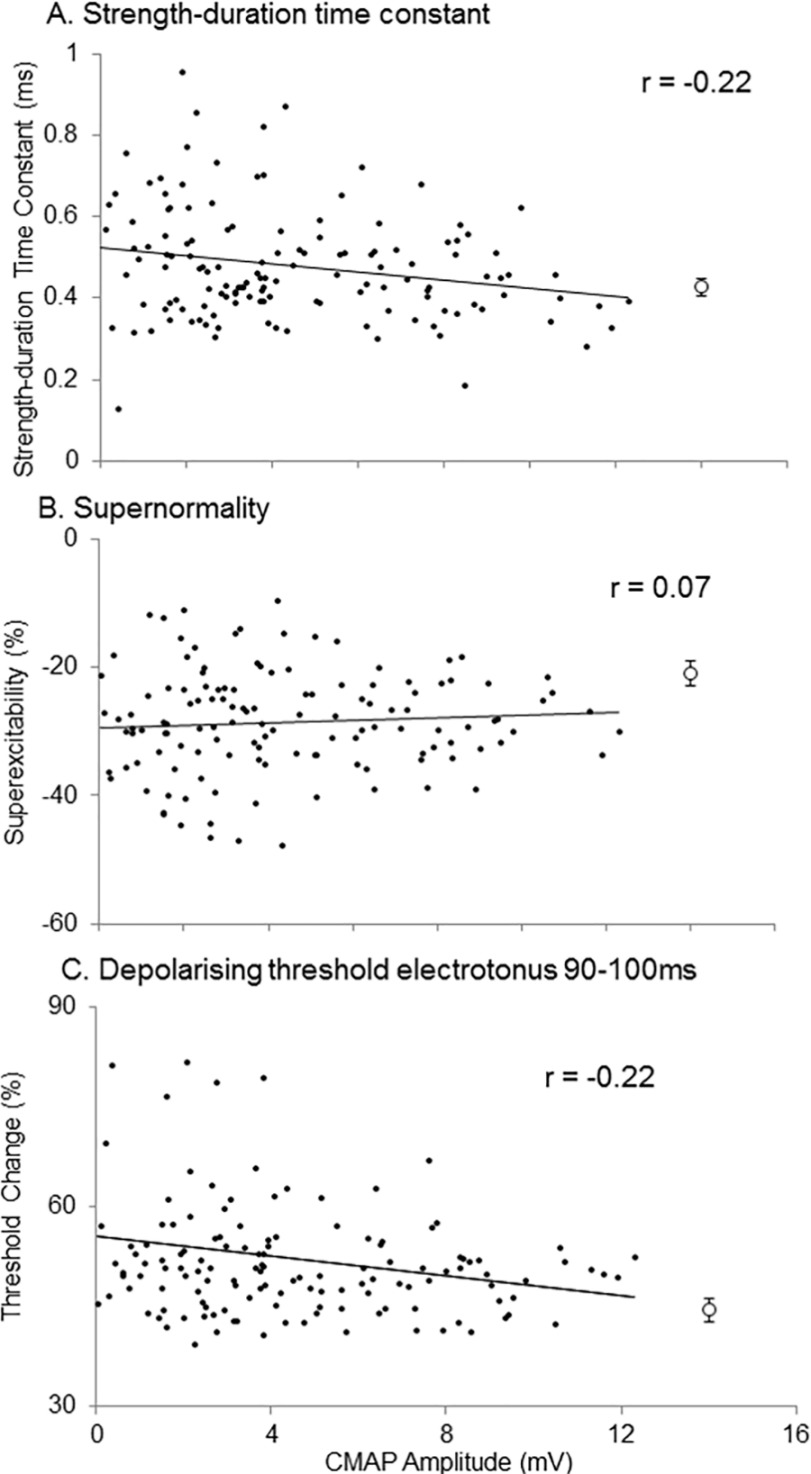
Scatter plots of nerve excitability indices and CMAP amplitudes. Scatter plots of nerve excitability indices and compound muscle action potential (CMAP) amplitudes in 140 ALS patients. These indices were recorded over the abductor pollicis brevis (APB) muscle, stimulated in the median nerve at the wrist. SDTC (A) (p = 0.01, r = -0.22), superexcitability (B) (p = 0.45, r = 0.067) and TEd 90-100ms (C) (p = 0.01, r = -0.22) were increased with CMAP decline. Approximate lines are shown in each index. These findings suggest increased persistent sodium and decreased potassium currents deteriorate with axonal loss. Open circles represent normal average ± 1.96SE value in each index.

Results of correlation analyses between CMAP amplitude and excitability indices are shown in Table 2 and Fig 2. SDTC (p = 0.01, R = -0.22) and TEd 90-100ms (p = 0.01, R = -0.22) were significantly related to CMAP amplitude and increased with CMAP decline. These findings suggest that increased persistent sodium and decreased slow potassium currents are significantly related to axonal loss and become prominent with axonal loss. TEd 10-30ms (R = -0.11) increased, and superexcitability (R = 0.067), late subexcitability (R = 0.16) and threshold in 40% depolarizing currents (R = -0.19) decreased with CMAP decline. These were not significantly associated with CMAP amplitude, but these alteration were consistent with the previous result, decreased potassium currents becoming prominent with CMAP decline [13].

**Table 2:** The correlation between nerve excitability indice and CMAP amplitude.

Nerve excitability indice			Results of correlation analyses	
Strength-duration time constant		(ms)	p = 0.01	R = -0.22
Threshold electrotonus				
	TEd (10–30 ms)	(%)	p = 0.19	R = -0.11
	TEd (90–100 ms)	(%)	p = 0.01	R = -0.22
	TEh (90–100 ms)	(%)	p = 0.67	R = -0.04
Recovery cycle				
	Refractoriness	(%)	p = 0.96	R = 0.004
	Superexcitability	(%)	p = 0.45	R = 0.067
	Late subexcitability	(%)	p = 0.07	R = 0.16
Current threshold relationship				
	40% depolarizing current	(%)	p = 0.03	R = -0.19
	100% hyperpolarizing current	(%)	p = 0.10	R = -0.15

TE = threshold electrotonus

## Discussion

Our 140 ALS patient data demonstrated increased persistent sodium conductances and decreased potassium currents in peripheral motor axons, suggesting motor axonal hyperexcitability. Reduced potassium currents were evident even in patients with preserved CMAP. Increased persistent sodium conductance and decreased slow potassium currents were significantly related to CMAP amplitude and deteriorate with axonal loss. These findings suggest motor axonal hyperexcitability precedes axonal loss.

Results of excitability testing demonstrated ALS patients had longer SDTC, greater TEd, greater superexcitability and reduced late subexcitability in recovery cycle and greater reduction of threshold in 40% depolarizing current in current-threshold relationship. Although there are various interpretation on these findings, multiple excitability measurements mainly assess axonal ion channel function, especially sodium and potassium currents, and membrane potential [16,17]. Our findings may be suitable for those of increased nodal persistent sodium currents, decreased juxtaparanodal fast potassium currents and decreased nodal slow potassium currents [5,6,8,17,20,21]. Results of mathmatical modeling in previous study, increased sodium and decreased potassium currents, pathological findings, decreased expression of potassium channel in ALS motor axon, and serological study, presence of anti-pottasium channel antibody in ALS patients, may support our interpretation [5,6,22]. We may have to retry mathematical modeling in the future study.

Our analyses suggested motor nerve hyperexcitability appears in patients with preserved CMAP. As described earlier, motor axonal hyperexcitability potentially contributes to motor neuron death, although the onset of this alteration has not been elucidated. Our findings suggest that nerve hyperexcitability, especially decreased potassium currents, precedes axonal loss. Peripheral nerve hyperexcitability may increases Ca^2+^ influx in lower motor neuron, lead to activation of degenerative enzymes, cause mitochondorial dysfunction, produce free radical, cause impaired production of adenosime triphosphate and result in motor neuron death [12]. As such, peripheral nerve hyperexcitability may be in the relatively upstream in the degenerative process of ALS neurodegeneration. While significantly decreased potassium currents were found in patients with preserved CMAP, increased persistent sodium currents were not. Potassium currents are measured using 3 parameters, TE, recovery cycle and current threshold relationship, although the parameters of persistent sodium currents are less. It is already reported that persistent sodium currents are strongly related to prognosis [23], suggesting that persistent sodium currents are potentially associated with motor neuron death in ALS and play an important role on pathogenic process. As such, further validations are necessary to conclude whether patietns with preserved CMAP really don’t have increased persistent sodium currents. In any case, motor axonal hyperexcitability, at least decreased potassium currents, may precede axonal loss. If nerve hyperexcitability contributes to degenerative pathway in ALS, ion channel modulators could be potential therapeutic drugs for ALS treatment [15].

Altered channel dysfunctions were more prominent in patients with axonal loss, compared to preserved axons. Additionally, these trends were also found in regression analyses of all nerve excitability measurements (SDTC, TE, recovery cycle and current threshold relationship). These results may suggest that axonal hyperexcitability becomes prominent with axonal loss. As noted previously, one longitudinal study revealed that potassium currents decrease with disease progression and are consistent with our findings [13]. Separately, our 58 ALS data previously demonstrated that increased sodium currents are found in patient with preserved CMAP, and decreased potassium currents are exhibited in patient with moderately decreased CMAP [5]. In the present study, our large cohort, including 58 previous data, might make it clearer. Several studies disclosed the relationship between ALS pathology and hyperexcitability [24–26]. One study demonstrated motor neuronal cell line, transfected with transactivation response element DNA binding protein 43 (TDP-43), has hyperexcitability [27]. Additionally, if ALS pathology, such as TDP-43, propagates through transcellular pathyway, “prion-like propagation”, ALS pathological changes may gradually lead motor neuronal hyperexcitability from cell to cell [28–30]. Separately, our prior autopsy study revealed markedly reduced potassium channel expression in ALS motor axon [6]. Moreover, prior electromyogram study revealed that fasciculation potential frequency does not change even when the number of motor units is significantly decreased [31]. These results and hypotheses may support our findings. However, further validation is necessary for our assumptions, because significant relationships between CMAP amplitude and excitability indices were found only in SDTC and TEd 90-100ms.

One limitation of this study is that CMAP amplitude was used as a marker of axonal loss. In the condition of axonal loss, reinnervations affect CMAP amplitude. Neurophysiological index, which is calculated from CMAP amplitude, distal motor latency and F-wave frequency, or motor unit number estimation may be more suitable for evaluating ALS axonal loss [32,33]. Furthermore, while we excluded patients with concomitant diseases which affect axonal functions, patients with mild and latent these diseases might be included. However, CMAP amplitude reflects and is significantly related to axonal loss, and our large cohort may diminish these limitations [33]. To validate our findings, longitudinal neurophysiological follow-up from the time of early disease stage may be helpful. Additionally, while we assessed axonal excitability in a large cohort, our excitability technique evaluates axonal function only at stimulation site, and that may have limitations.

This study suggests that nerve hyperexcitability is early features. Nerve hyperexcitability may induce motor neuron death and accelerate motor neuron death.

## Supporting Information

S1 Table Raw data of 140 ALS patients. (XLSX)
